# Mediators, Receptors, and Signalling Pathways in the Anti-Inflammatory and Antihyperalgesic Effects of Acupuncture

**DOI:** 10.1155/2015/975632

**Published:** 2015-08-03

**Authors:** John L. McDonald, Allan W. Cripps, Peter K. Smith

**Affiliations:** School of Medicine, Griffith Health Institute, Griffith Health, Griffith University, Southport, QLD 4211, Australia

## Abstract

Acupuncture has been used for millennia to treat allergic diseases including both intermittent rhinitis and persistent rhinitis. Besides the research on the efficacy and safety of acupuncture treatment for allergic rhinitis, research has also investigated how acupuncture might modulate immune function to exert anti-inflammatory effects. A proposed model has previously hypothesized that acupuncture might downregulate proinflammatory neuropeptides, proinflammatory cytokines, and neurotrophins, modulating transient receptor potential vallinoid (TRPV1), a G-protein coupled receptor which plays a central role in allergic rhinitis. Recent research has been largely supportive of this model. New advances in research include the discovery of a novel cholinergic anti-inflammatory pathway activated by acupuncture. A chemokine-mediated proliferation of opioid-containing macrophages in inflamed tissues, in response to acupuncture, has also been demonstrated for the first time. Further research on the complex cross talk between receptors during inflammation is also helping to elucidate the mediators and signalling pathways activated by acupuncture.

## 1. Introduction

In our previous publication, research on the anti-inflammatory effects of acupuncture was reviewed and a model was proposed to guide further research [1]. This review included both demonstrated and proposed effects of acupuncture and drew from both animal and human studies. Anti-inflammatory effects of acupuncture in contexts other than allergic rhinitis were explored for their potential relevance to allergic rhinitis, especially research involving proinflammatory neuropeptides and neurotrophins and modulation of the transient receptor potential vallinoid 1 (TRPV1), a central receptor in allergic inflammatory response.

New research has expanded and clarified the understanding of inflammatory response and how acupuncture might modulate it, and hence a revised model for the anti-inflammatory effects of acupuncture is proposed.

## 2. The Previously Proposed Model (2013 Model)

Our 2013 model proposed that, in allergic rhinitis, acupuncture downregulated proinflammatory neuropeptides, substance P (SP), calcitonin gene-related peptide (CGRP) and vasoactive intestinal peptide (VIP); downregulated neurotrophins, nerve growth factor (NGF), and brain-derived neurotrophic factor (BDNF); and downregulated Th2 and proinflammatory cytokines and possibly upregulated Th1 cytokines, thereby shifting Th1/Th2 balance away from Th2 dominance [1]. Acupuncture-induced modulation of TRPV1 (both demonstrated and hypothesised) was also explored.

## 3. Effects of Acupuncture on Inflammatory Oedema

Inflammatory oedema has been significantly reduced by acupuncture in rodent and murine models of induced hind paw inflammation [2, 3]. This antioedema effect is mediated via the HPA axis as various disruptions of the HPA axis obliterate or significantly attenuate the effect [2, 3]. Many of the pathways, mediators, and receptors involved in acupuncture attenuation of inflammatory hyperalgesia appear to have either no effect or delayed effects on inflammatory oedema [4, 5]. For example, opioid pathways are implicated in acupuncture effects on inflammatory hyperalgesia but have generally been shown to have no influence on inflammatory oedema as opioid antagonists did not diminish the antioedema effect [4, 6–8]. One study, however, has shown slight reduction in inflammatory oedema which was blocked by opiate antagonists naloxone and natrindole [9].

A single acupuncture treatment was previously reported to produce a significant but short-lived (less than 15 minutes) improvement in the patency of the nasal airway [28]. However, subjective sensations of nasal congestion have consistently been reported to decrease significantly for much longer periods following acupuncture for allergic rhinitis in adults [29–33].

Swelling of the nasal mucosa in allergic rhinitis has been linked to proinflammatory neuropeptides, proinflammatory cytokines, neurotrophins, and chemokines [34].

### 3.1. Neuropeptides

Downregulation of proinflammatory neuropeptides, SP, and VIP, after EA, has previously been reported in adults with persistent allergic rhinitis [35]. SP was also found to decrease sharply (by 83.2%) 18 to 24 hours after the first manual acupuncture treatment in adults with persistent allergic rhinitis in a study conducted by the authors (unpublished data). Substance P expression was also inhibited in the nasal mucosa of mice with experimentally induced allergic rhinitis following acupuncture (associated with inhibition of signal transducer and activator of transcription 6 (STAT 6), nuclear factor kappa B (NF*κ*B), and inducible nitric oxide synthase (iNOS)) [36]. While CGRP downregulation after acupuncture has been reported in migraine, menopausal hot flushes, and spinal nerve lesion studies, to date no studies have shown a downregulation of CGRP in allergic rhinitis.

### 3.2. Cytokines

STAT 6 and NF*κ*B are transcription factors which play an essential role in Th2 cell differentiation [36]. Inhibition of STAT 6 and NF*κ*B would therefore suppress Th2 cell differentiation, shifting the Th1/Th2 balance away from Th2 dominance, which characterizes atopy [36]. Significant decreases in STAT 6 and NF*κ*B, along with the vasodilator iNOS were reported in the mouse study mentioned in the previous paragraph [36]. A recent human study comparing acupuncture with Loratadine for perennial allergic rhinitis reported an increase in mean values for interleukin-10 (IL-10), but the increase was not statistically significant, possibly due the study being underpowered [37]. The same study also found no change in allergen-specific immunoglobulin E (IgE) for house dust mite, total IgE, interleukin-4 (IL-4), or interferon gamma (IFN-*γ*) [37]. A small number of earlier human studies on the treatment of allergic rhinitis with acupuncture showed that a trend towards Th2 cytokines os being downregulated, but very little evidence of Th1 cytokines is being upregulated [31, 38–40].

### 3.3. Neurotrophins

Neurotrophins have been shown to be selectively upregulated or downregulated by acupuncture depending on the condition. Recent research on neurotrophins has mainly focused on cerebral ischaemia, spinal cord injury, and depression [13, 41–44]. No research has yet been published on the possible modulation of neurotrophins by acupuncture in allergic rhinitis. It is hypothesised, however, that the abundance of VIP+ parasympathetic nerves around blood vessels in the lamina propria of the nasal mucosa (which has been observed in allergic rhinitis) may be the result of upregulation of neurotrophins, and this neuronal abundance would contribute to exacerbating inflammatory oedema in the nasal mucosa [45–47].

### 3.4. Chemokines

Chemokines have received little attention from acupuncture researchers until very recently. A new study has reported that EA upregulated chemokine CXCL10 in a rodent model of complete Freund's adjuvant- (CFA-) induced hind paw inflammation [16]. EA increased the production and release of both IFN-*γ* and CXCL10 which in turn increased the number of infiltrating opioid peptide-containing CXCR3+ macrophages [16]. This study establishes an important novel link between immune system mediators such as cytokines, chemokines, and macrophages and the nervous system's opioid pathways.

The effects of acupuncture on chemokines and chemokine receptors appear to be selective. The upregulation of CXCL10 increased the production and release of CXCR3+ macrophages which contain opioids [16]. Activation of opioid receptors, in turn, will desensitize chemokine receptors thereby inhibiting the production and release of proinflammatory cytokines as well as the chemokines CCL2, CCL3, and CXCL8 [48]. In a rat model of embryo implantation failure, acupuncture increased expression of CCL2, CXCL8, and the subset of uterine natural killer (uNK) cells in the endometrium and reduced embryo implantation failure [49].

## 4. Complex Cross Talk between Receptors

**Figure 1:**
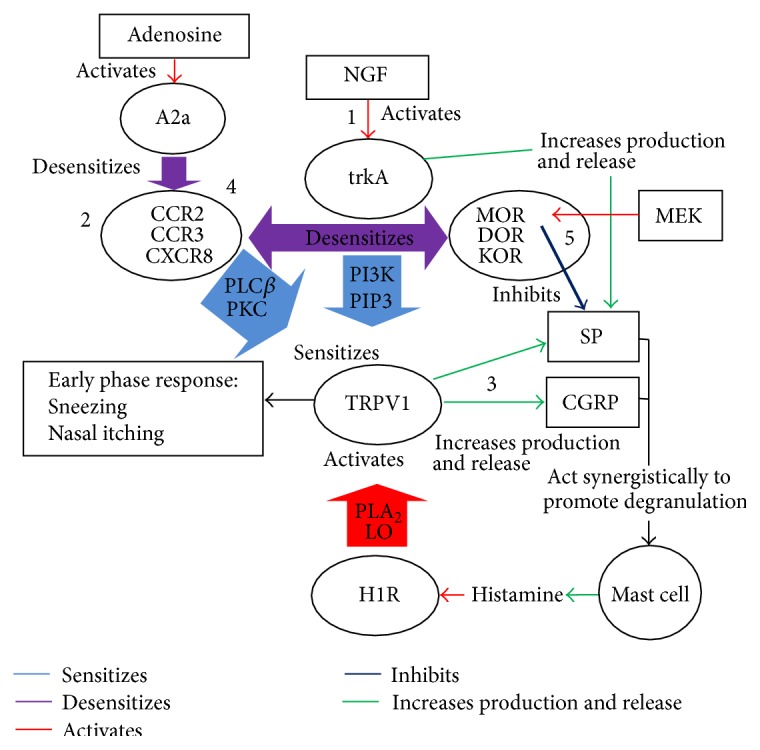
Proposed model for the complex cross talk between various receptors and mediators in early phase response in allergic rhinitis. 1: nerve growth factor (NGF) activates tyrosine kinase A (TrkA) receptor which in turn increases production and release of substance P (SP). Activation of TrkA receptor also initiates signalling via the PI3K/PIP3 pathway to increase expression and sensitivity of transient receptor potential vallinoid (TRPV1) receptor. 2: chemokine receptors (CCR2, CCR3, and CXCR8) sensitize TRPV1 receptor via a PLC*β*/PKC pathway. 3: TRPV1 receptor increases production and release of proinflammatory neuropeptides SP and CGRP which act synergistically to promote degranulation of primed mast cells. Histamine released by mast cells activates histamine 1 receptor (H1R) producing signalling via the phospholipase A_2_/lipoxygenase pathway to activate TRPV1, triggering early phase allergic inflammatory response. 4: chemokine receptors are heterologously desensitized by both adenosine (A2a) receptors and opioid receptors (MOR, DOR, and KOR). 5: Substance P is inhibited by met-enkephalin via Mu opioid receptors (MOR). A2a: adenosine 2a receptor, CCR2, CCR3: CC chemokine receptors 2 & 3, CXCR8: CXC chemokine receptor 8, PLC*β*: phospholipase C *β*, PKC: protein kinase C, NGF: nerve growth factor, TRPV1: transient receptor potential vallinoid 1, TrkA: tyrosine kinase A receptor, H1R: histamine 1 receptor, SP: substance P, CGRP: calcitonin gene-related peptide, PI3K/PIP3: phosphatidylinositol 3 kinase/phosphatidylinositol phosphate 3 pathway, PLA_2_/LO: phospholipase A_2_/lipoxygenase pathway, MOR: Mu opioid receptor, DOR: delta opioid receptor, KOR: kappa opioid receptor, and MEK: met-enkephalin.

Chemokine receptors, opioid receptors and TRPV1 receptors have been reported to participate in complex cross talk, not unlike that between neuropeptides, cytokines, and neurotrophins [48] Chemokine receptors and opioid receptors can inhibit each other by a process known as heterologous desensitization [48]. Adenosine, by activating A2a receptors, can also desensitize chemokine receptors [48] (see Figure 1). Chemokine receptors sensitize TRPV1; hence, any desensitization of chemokine receptors (by adenosine or opioid receptors) would inhibit the sensitization of TRPV1 (via a phospholipase C *β*/protein kinase C [PLC*β*/PKC] pathway) [48]. Interactions between cannabinoid receptors and opioid receptors after acupuncture have been reported. Cannabinoid receptor CB2 has been shown to participate in the EA-induced increase of opioid expression in keratinocytes in inflamed skin (in a CFA rat hind paw inflammation model) [12].

Cannabinoid receptor CB1 has been shown to mediate phosphorylation of both STAT3 and glycogen synthase kinase-3*β* after acupuncture in rodent cerebral ischaemia studies [50, 51].

Upregulation of CB1 expression after EA also produced upregulation of dopamine receptors D1 and D2 in the striatum (in a CFA rat hind paw inflammation model) [14].

Since acupuncture has already been shown to be capable of activating adenosine and opioid receptors and now has been shown to selectively modulate chemokines, there are a number of possible pathways via which TRPV1 might be downregulated by acupuncture.

EA has previously been reported to downregulate TRPV1 by inhibiting phosphorylation of PI3K in the dorsal horn of rats (in a carrageenan hind paw inflammation model) [19]. NGF activates the tyrosine kinase A/phosphatidylinositol 3-kinase/phosphatidylinositol phosphate 3/protein kinase Akt (trkA/PI3K/PIP3/Akt) signalling pathway which increases the sensitivity and expression of TRPV1, so blocking this pathway would prevent this sensitization of TRPV1 by NGF [19]. EA also activated p38 mitogen activated protein kinase/activating transcription factor 2/transient receptor potential vallinoid (p38 MAPK/ATF-2/TRPV1) signalling pathway thereby downregulating TRPV1 expression in the dorsal horn of rats; however, cyclooxygenase 2 (COX-2) was found to play no role (in a CFA hind paw inflammation model) [5]. In the same study, hind paw oedema was unchanged until day 14 after CFA injection. However, the reasons for this delayed action are unclear [5].

## 5. Effects of Acupuncture on Thermal Hyperalgesia and Mechanical Allodynia in Inflammatory and Neuropathic Pain

According to the ancient Roman doctor Celsius, inflammation was characterised by redness, heat, swelling, and pain [52]. In research using animal inflammatory pain models, pain threshold is generally measured as a response to mechanical pressure (allodynia) or to changes in temperature, usually heat (thermal hyperalgesia). In reducing inflammatory hyperalgesia, low frequency EA (typically 1 to 4 Hz) has been consistently reported to be effective (see Table 1). However, there is conflicting data on the effectiveness of high frequency EA (100–150 Hz) with some researchers reporting that 120 Hz EA failed to alleviate thermal hyperalgesia and mechanical allodynia in diabetic neuropathic pain in rats, while 2 Hz EA was effective [22]. Other studies have reported effective attenuation of inflammatory hind paw hyperalgesia in rats using 100 Hz EA [11, 14, 16].

Low frequency EA (2 Hz) effectively relieved mechanical allodynia in a rat spinal nerve ligation model [23]. This improvement in allodynia was associated with a reduction of both TRPV1 and CGRP in the ipsilateral spinal dorsal horn immediately above and below the lesioned level (L5) [23]. Since TRPV1 is capable of producing and releasing both CGRP and SP, the decrease in CGRP may be a direct result of the reduced expression of TRPV1 [53]. Additionally, since CGRP and SP interpromote each other, the CGRP downregulation could be secondary to a downregulation of SP production and release by TRPV1 [54].

EA effects on thermal hyperalgesia were blunted, but not obliterated, in TRPV1 knock-out mice [55]. TRPV1 knock-out mice required a stronger EA stimulation than wild type (C 57 BL/6) mice to achieve an analgesic effect [55]. This suggests that while TRPV1 downregulation contributes to EA effects on thermal hyperalgesia, TRPV1 is not solely responsible, as TRPV1 knock-out did not obliterate the effect. In contrast, histamine-induced itch is completely extinguished in TRPV1 knock-out mice [56].

Low frequency EA (2 Hz) has also been shown to decrease mechanical hyperalgesia in both CFA and carrageenan-induced CD1 mouse hind paw inflammation, accompanied by a significant downregulation of acid sensing ion channel 3 (ASIC3) overexpression in dorsal root ganglion neurons [17]. In another study, also using CFA and carrageenan-induced mouse hind paw inflammation (this time in ICR mice), 2 Hz EA reduced mechanical and thermal hyperalgesia accompanied with decreased expression of sodium voltage-gated (Nav) channels Nav1.7 and Nav1.8 (but not Nav1.9) in dorsal root ganglion neurons [18].

In another CFA hind paw inflammation study with rats, EA alternating between 2 Hz and 100 Hz alleviated mechanical allodynia and blocked activation of the extracellular-regulated protein kinase 1/2-cyclooxygenase-2 (ERK1/2-COX-2) and extracellular-regulated protein kinase 1/2-cAMP response element binding protein- neurokinin-1 receptor (ERK1/2-CREB-NK-1) pathways, but had no effect on Ets-like kinase 1 (Elk1) (a downstream nuclear substrate of ERK1/2) in the spinal dorsal horn [15]. Since SP is the endogenous ligand for NK-1 receptor, blocking NK-1 would downregulate the proinflammatory effects of SP, as has been previously reported [57].

In a spinal cord injury neuropathic pain model in rats, manual acupuncture significantly relieved thermal hyperalgesia and mechanical allodynia [24]. These acupuncture analgesic effects were associated with Jun-N-terminal kinase (JNK) inhibition in astrocytes in lamina I-II of the dorsal horn and inhibited phosphorylation of JNK downstream substrate, c-Jun [24]. Acupuncture also inhibited JNK-dependent expression of chemokines monocyte chemotactic protein 1 (MCP-1) (also known as CCL2), macrophage inflammatory protein 1*β* (MIP-1*β*) (also known as CCL4), and macrophage inflammatory protein 3*α* (MIP-3*α*) (also known as CCL20) in spinal astrocytes [24]. In a rat CFA hind paw study, EA alleviated mechanical allodynia (alternating between 2 Hz and 100 Hz) but not paw oedema by suppressing the spinal JNK1/2 pathway [4]. In this study, TRPV1 expression in the spinal dorsal horn was unaffected [4].

Further research is needed to investigate whether these modulations of TRPV1, produced by acupuncture in the spinal dorsal horn of rats or mice, can also be found in the trigeminal ganglia or nasal mucosa of humans with allergic rhinitis.

## 6. A Novel Cholinergic Anti-Inflammatory Pathway

It has been suggested that acupuncture may activate a cholinergic anti-inflammatory pathway involving acetylcholine release from vagus nerves binding to *α*7-nicotinic receptors (*α*7-nAChRs) on macrophages, thereby inhibiting the release of proinflammatory cytokines [58]. A recent study by Torres-Rosas et al. has demonstrated that there is in fact an anti-inflammatory pathway activated by EA which involves the vagus and sciatic nerves and is mediated by dopamine [59]. However, this study also found that *α*7-nAChRs were not involved in this anti-inflammatory pathway [59]. In a mouse model of induced sepsis, EA rescued mice from polymicrobial peritonitis and controlled systemic inflammation [59]. EA stimulation of the sciatic nerve induced release of DOPA decarboxylase from the vagus nerve leading to dopamine production in the adrenal medulla [59]. Of the dopamine receptor family, D1 receptors were shown to be essential to EA anti-inflammatory effects, but D2 receptors appeared to have little involvement [59]. Vagal stimulation was also associated with reduced serum levels of the cytokines tumour necrosis factor (TNF), MCP-1/CCL2, IL-6, and IFN-*γ* [59]. TRPV1 agonist, capsaicin, abolished the anti-inflammatory effects of EA [59]. This underlines the importance of TRPV1 role in the anti-inflammatory effects of acupuncture.

## 7. Adenosine

Adenosine triphosphate (ATP) released locally at acupuncture points, in response to needle stimulation is metabolized into adenosine, which activates adenosine A1 receptors, creating an analgesic effect in inflammatory pain [60, 61]. Adenosine, via A2a receptors, has also been shown to desensitize chemokine receptors, hence blocking the capacity of chemokine receptors to both sensitize TRPV1 receptors via a PLC*β*/PKC pathway and to desensitize opioid receptors [48]. Adenosine has previously been reported to inhibit TRPV1 activation, and this action may be mediated via the PLC*β*/PKC pathway [62]. In addition, adenosine, via A3 receptor activation has recently been shown to be effective in relieving persistent neuropathic pain [63]. Further investigation is needed to determine whether or not adenosine modulates TRPV1 (and possibly opioid receptors) in the nasal mucosa or trigeminal ganglia.

## 8. A Revised Model for the Effects of Acupuncture in Allergic Rhinitis

The central importance of TRPV1 in allergic rhinitis raises the question of how acupuncture might modulate TRPV1 either directly or indirectly. In our previous proposed model, it was suggested that acupuncture might modulate TRPV1 by blocking neurotrophic sensitization (by blocking phosphorylation of PI3K, as seen in EA for neuropathic pain), by downregulating SP and CGRP (with consequent suppression of degranulation of primed mast cells) and possibly through some pathway associated with adenosine. It now appears that this adenosine-induced downregulation of TRPV1 may occur through desensitization of chemokines receptors (which sensitize TRPV1 via a PLC*β*/PKC pathway) [48] (see Figure 1).

Downregulation of SP by met-enkephalin has previously been reported [64, 65]. New research showing that acupuncture can stimulate production of CXCR3+ macrophages (which contain met-enkephalin) clarifies the source of the opioid stimulation. Opioid receptor desensitization of chemokine receptors, with consequent inhibition of both cytokine production and sensitization of TRPV1, provides another pathway via which acupuncture-induced opioid stimulation could generate anti-inflammatory effects.

TRPV1 interactions with both cannabinoid and dopamine receptors have also now been demonstrated in murine and rodent studies of inflammatory hyperalgesia (see Table 1). Further studies are needed to determine whether these effects of acupuncture in the spinal dorsal root ganglia during inflammatory hyperalgesia are relevant to the trigeminal ganglia in allergic rhinitis.

Recent research into the effects of acupuncture on signalling pathways and receptors in inflammatory pain has focused mainly on mechanical allodynia and thermal hyperalgesia, but little new work has been done on inflammatory oedema. Since TRPV1 appears to play a central role in inflammatory oedema, it is possible that signalling pathways activated by acupuncture which are independent of TRPV1 may be associated with reduction of thermal and mechanical hyperalgesia but not inflammatory oedema [66]. Since nasal swelling is more significant clinically in allergic rhinitis than hyperalgesia, signalling pathways identified in hyperalgesia studies which do not involve TRPV1 may be less relevant to acupuncture's effects on allergic rhinitis.

The effects of acupuncture on chemokines and chemokine receptors appear to be selective. While desensitization of chemokine receptors by adenosine and by interaction with opioid receptors may reduce sensitivity of TRPV1, it is also clear that acupuncture can increase chemokines such as CXCL10, CCL2, and CXCL8.

**Figure 2:**
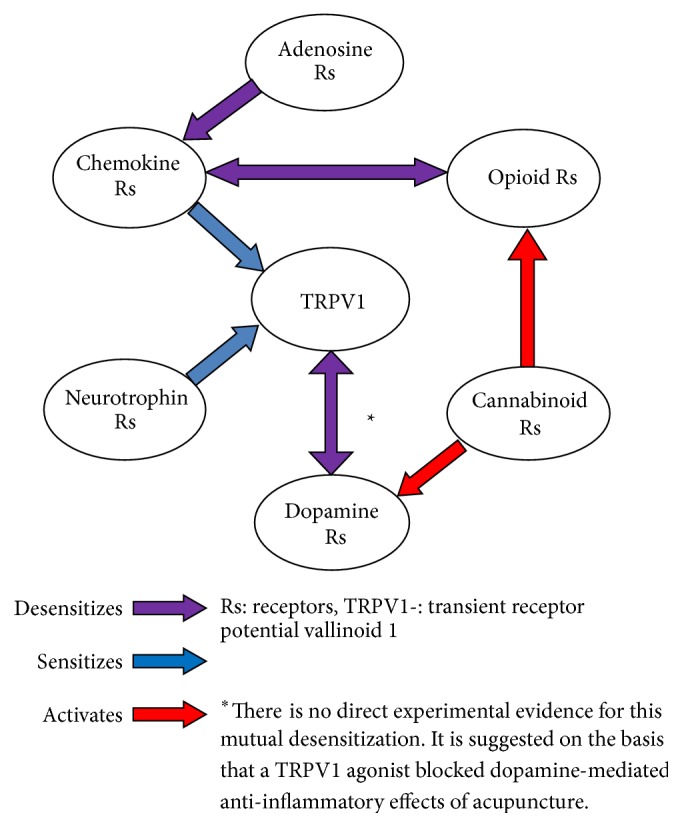
Receptor interactions potentially involved in the anti-inflammatory effects of acupuncture.

Interactions between receptor types are now being reported with an increasing frequency. Acupuncture has been shown to influence the interactions between opioid receptors and chemokine receptors, as well as cannabinoid receptors (see Figure 2). Interactions between cannabinoid receptors and dopamine receptors have also been shown to be modulated by acupuncture [14].

## 9. Conclusion

Our 2013 model proposed that acupuncture downregulates proinflammatory neuropeptides and neurotrophins, alters Th1/Th2 cytokine balance, and modulates TRPV1.

Acupuncture has already been shown to downregulate SP and VIP in allergic rhinitis and may also downregulate CGRP. This reduction in SP and VIP has been associated with improvements in clinical signs and symptoms.

Acupuncture may downregulate neurotrophins NGF and BDNF in allergic rhinitis, but evidence is currently lacking.

Acupuncture has been shown to alter Th1/Th2 cytokine balance away from Th2 dominance in a variety of clinical contexts including allergic rhinitis; however, more robust evidence is still needed. The role of transcription factors involved in Th2 differentiation (such as STAT 6 and NF*κ*B) merits further investigation.

While numerous new signalling pathways have been identified in the anti-inflammatory actions of acupuncture in animal model studies on induced hind paw inflammation, spinal cord injury, and chronic constriction injury, it is unclear how much relevance these studies might have to allergic rhinitis (see Table 1). Frequently, the main outcome measures for these studies have been measures of inflammatory pain, thermal and mechanical hyperalgesia, as opposed to inflammatory oedema, which would be more likely to be relevant to allergic rhinitis.

Recent research has also shown that acupuncture can modulate chemokines and that interactions between chemokine and opioid receptors may have relevance to reducing the expression and sensitivity of TRPV1. Further research on the effects of acupuncture on chemokines is needed.

A novel cholinergic anti-inflammatory pathway involving vagal stimulation and dopamine mediation has now been demonstrated [59]. This pathway is neither parasympathetic nor sympathetic but involves both. In acupuncture analgesia research, dopamine receptors have been shown to act in a selective manner. D1 receptors in the brain inhibited acupuncture analgesia, while D2 receptors in the spine enhanced analgesic effects [27]. Torres-Rosas et al. demonstrated that D1 receptors appeared to play a significant role in the anti-inflammatory effects of acupuncture while D2 receptors did not [59]. The role of dopamine receptors and their interactions with cannabinoid receptors, and possibly TRPV1, in the anti-inflammatory effects of acupuncture have only just begun to be explored. Further exploration of receptor interactions and their possible involvement in acupuncture effects would be valuable.

## Figures and Tables

**Table 1:** Effects of electroacupuncture and manual acupuncture on inflammatory signalling pathways and receptors.

Author and year	Model	EA/acup	Acupuncture points	Effect	Thermal hyperalgesia	Mechanical allodynia	Oedema
Huang et al. 2004 [10]	CFA hind paw FSD rats	EA 100 Hz	(B) ST 36, SP 6		No effect	Reduced	

Huang et al. 2008 [11]	CFA hind paw FSD rats	EA 100 Hz	(B) ST 36, SP 6	Reduced thermal hyperalgesia and increased EA tolerance EA effects abolished by high dose Naloxone suggesting dynorphin mediation	Reduced		

Su et al. 2011 [12]	CFA hind paw MSD rats	EA 2 Hz	(I) GB 30, GB 34	Increased POMC and *β*-END expression in keratinocytes, macrophages, and T-lymphocytes in inflamed skin via activation of CB2 cannabinoid receptors	Reduced	Reduced	

Zhang et al. 2012 [13]	CFA hind paw rats	EA 10 Hz	GB 30	Suppressed spinal IL-17 and p-NR1	Reduced	Reduced	

Shou et al. 2013 [14]	CFA hind paw MSD rats	EA 2 Hz EA 100 Hz	ST 36, BL 60	Increased CB1 expression with upregulation of D1 and D2 expression in striatum	Reduced		

Fang et al. 2013 [5]	CFA hind paw MSD rats	EA 2/100 Hz alt	(B) ST 36, BL 60	Inhibition of p38/MAPK/ATF-2/TRPV1 pathway producing downregulation of TRPV1 in spinal dorsal horn		Reduced	Reduced only at day 14

Fang et al. 2014 [15]	CFA hind paw MSD rats	EA 2/100 Hz alt	(B) ST 36, BL 60	Inhibited ERK1/2-COX-2 and ERK1/2-CREB-NK-1 pathway Downregulated NK-1 hence inhibiting SP		Reduced	

Wang et al. 2013 [9]	CFA hind paw M Wistar rats	EA 100 Hz	(B) GB 30	EA effects suppressed by opiate antagonists naloxone and natrindole	Reduced	Reduced	Reduced slightly

Wang et al. 2014 [16]	CFA hind paw M Wistar rats	EA 100 Hz	(B) GB 30	TNF-*α* and IL-1*β* downregulated, IL-13 upregulated, and IL-1*α* and IL-4 unchanged IFN-*γ* up-regulated stimulating CXCL10 increasing CXCR3+ macrophages	Reduced	Reduced	

Du et al. 2014 [4]	CFA hind paw MSD rats	EA 2/100 Hz alt	(B) ST 36, BL 60	Inhibited JNK1/2 and COX-2 but not TRPV1		Reduced	No effect

Chen et al. 2011 [17]	Carrageenan & CFA hind paw CDI mice	EA 2 Hz	ST 36	Decreased overexpression of ASIC3 in DRG		Reduced	

Huang et al. 2013 [18]	Carrageenan & CFA hind paw F ICR mice	EA 2 Hz	ST 36	Decreased expression of sodium voltage-gated channels Nav 1.7 and Nav 1.8 but not Nav 1.9 in DRG	Reduced	Reduced	

Kim et al. 2012 [19]	Carrageenan hind paw MSD rats	EA 2/100 Hz alt	(B) ST 36, SP 6	Inhibition of p-PI3K blocked trkA/PI3K/PIP3/Akt pathway	Reduced	Reduced	

da Silva et al. 2015 [20]	Carrageenan gastrocnemius C57BL/6 mice	Manual acup	SP 6	Increased IL-10 in inflamed muscle Induced a phenotypic switch from M1 to M2 macrophages in inflamed muscle	Reduced	Reduced	Reduced

Kim et al. 2009 [21]	Capsaicin hind paw MSD rats	EA 2 Hz	(I) SI 3, TE 8 (GB 30, GB 34; BL 40, BL 60; GV 2, GV 6; LI 3, LI 6)	SI 3 & TE 8 effective, but other point combinations were not Secondary (but nor primary) hyperalgesia is mediated by MOR and DOR but not KOR or adrenergic receptors		Reduced secondary but not primary hyperalgesia	

Hwang et al. 2011 [22]	Exp. diabetic neuropathic pain MSD rats	EA 2 Hz EA 120 Hz	(B) SP 9 or ST 36	Decreased cleavage of p35 to p25 hence inhibited p35/p25/Cdk5/MAPK and/or p35/p25/Cdk5/NMDA pathways	EA 2 Hz reduced; EA 120 Hz no effect	EA 2 Hz reduced; EA 120 Hz no effect	

Jiang et al. 2013 [23]	SNL MSD rats	EA 2 Hz	(I) ST 36, BL 60	Downregulated TRPV1 in spinal dorsal horn and reduced CGRP		Reduced	

Lee et al. 2013 [24]	SCI MSD rats	Manual acup	GV 26, (B) GB 34	Inhibited JNK/p-c-Jun in spinal astrocytes Decreased chemokines MCP-1, MIP-1*β*, and MIP-3*α*	Reduced	Reduced	

Yu et al. 2013 [25]	CCI SD rats	EA 2 Hz EA 15 Hz	ST 36, GB 34	EA reduced ERK1/2 phosphorlylation and P2X3 expression in spinal cord	Reduced EA 2 Hz > 15 Hz	Reduced EA 2 Hz > 15 Hz	

Hsu et al. 2014 [26]	CCI MSD rats	EA 2 Hz EA 15 Hz	(R) ST 36, ST 37	EA increased cerebral TRPV4 but not TRPV1 No change in spinal TRPV4 or TRPV1	Reduced	Reduced	

Wang et al. 2009 [27]	Surgical trauma MSD rats	EA 2/60 Hz alt	(B) ST 36, Lanwei (M-LE-13)	Suppressed lymphocyte proliferation Reduced splenic T cells production of Th1 cytokines (IL-2 and IFN-*γ*) increased Th2 cytokines (IL-4, IL-10) Suppressed activity of ERK1/2, p38, NF-*κ*B, and AP-1	Reduced	Reduced	

EA: electroacupuncture, acup: acupuncture, CFA hind paw: model of inflammation induced by injection of complete Freund's adjuvant into rats' hind paws, FSD rats: female Sprague Dawley rats, MSD: male Sprague Dawley rats, alt: alternating, (B): bilateral, (I): ipsilateral, CB: cannabinoid receptor, D: dopamine receptor, TRPV: transient receptor potential vallinoid, proopiomelanocortin, *β*-END: beta endorphin, p-NR1: phosphorylation of NR1, SP: substance P, CGRP: calcitonin gene-related peptide, NK-1: neurokinin 1 receptor, DRG: dorsal root ganglion, MAPK: mitogen activated protein kinase, ATF-2: activating transcription factor 2, ERK: extracellular-regulated protein kinase, CREB: cAMP response element binding protein, NK-1: neurokinin 1 receptor, JNK: c-jun N terminal kinase, ASIC3: acid sensing ion channel 3, Nav: sodium voltage-gated channels, trkA: tyrosine kinase A, PI3K/PIP3: phosphatidylinositol 3 kinase/phosphatidylinositol phosphate 3 pathway, MOR: Mu opioid receptor, DOR: delta opioid receptor, KOR: kappa opioid receptor, AP-1: activator protein 1, Cdk5: cyclin-dependent kinase 5, and NMDA: N-methyl-D-aspartate.
